# Evidence for the Role of *CYP51A* and Xenobiotic Detoxification in Differential Sensitivity to Azole Fungicides in Boxwood Blight Pathogens

**DOI:** 10.3390/ijms22179255

**Published:** 2021-08-26

**Authors:** Stefanos Stravoravdis, Robert E. Marra, Nicholas R. LeBlanc, Jo Anne Crouch, Jonathan P. Hulvey

**Affiliations:** 1 Department of Microbiology, University of Massachusetts Amherst, Amherst, MA 01003, USA; sstravoravdi@umass.edu; 2 Biology Department, Eastern Connecticut State University, Willimantic, CT 06226, USA; 3 Department of Plant Pathology and Ecology, The Connecticut Agricultural Experiment Station, New Haven, CT 06504, USA; Robert.Marra@ct.gov; 4 Mycology and Nematology Genetic Diversity and Biology Laboratory, United States Department of Agriculture, Agricultural Research Service, Beltsville, MD 20705, USA; nicholas.leblanc@usda.gov (N.R.L.); joanne.crouch@usda.gov (J.A.C.); 5 ARS Research Participation Program, Oak Ridge Institute for Science and Education, Oak Ridge, TN 37831-0117, USA

**Keywords:** *Calonectria*, ergosterol, demethylation inhibitors, azole, xenome, pseudogene

## Abstract

Boxwood blight, a fungal disease of ornamental plants (*Buxus* spp.), is caused by two sister species, *Calonectria pseudonaviculata* (*Cps*) and *C. henricotiae* (*Che*). Compared to *Cps*, *Che* is documented to display reduced sensitivity to fungicides, including the azole class of antifungals, which block synthesis of a key fungal membrane component, ergosterol. A previous study reported an ergosterol biosynthesis gene in *Cps*, *CYP51A*, to be a pseudogene, and RNA-Seq data confirm that a functional *CYP51A* is expressed only in *Che*. The lack of additional ergosterol biosynthesis genes showing significant differential expression suggests that the functional *CYP51A* in *Che* could contribute to reduced azole sensitivity when compared to *Cps*. RNA-Seq and bioinformatic analyses found that following azole treatment, 55 genes in *Cps*, belonging to diverse pathways, displayed a significant decrease in expression. Putative xenobiotic detoxification genes overexpressed in tetraconazole-treated *Che* encoded predicted monooxygenase and oxidoreductase enzymes. In summary, expression of a functional *CYP51A* gene and overexpression of predicted xenobiotic detoxification genes appear likely to contribute to differential fungicide sensitivity in these two sister taxa.

## 1. Introduction

Boxwood blight is a disease of native and commercially grown boxwood (*Buxus* spp.) caused by two emerging fungal plant pathogens: *Calonectria pseudonaviculata* (*Cps*) and *C. henricotiae* (*Che*) [1,2]. During infection, plants undergo stem and foliar lesioning, rapid defoliation, and die back, and preventative fungicides are required for disease management. *Cps* infects *Buxus* plants globally, while *Che* is restricted to Europe [1,2,3,4]. Both species were previously classified as distinct genotypes of *Cps*, but multi-locus phylogenetic analysis, comparative genomics, and phenotypic differences supported their placement into separate sister species, with the erection of *Che* [3,5].

Various classes of fungicides and antifungal drugs are used to control plant and animal diseases caused by fungal pathogens, including boxwood blight [6,7,8]. One group of antifungals, the azole class (or demethylation inhibitors/DMIs), impede production of ergosterol, a vital component of fungal cell membranes [9,10]. This class of antifungal chemicals has broad application in both agriculture and medicine and is used to treat and prevent a broad range of plant and animal diseases. Azoles inhibit fungal growth by affecting membrane integrity and are targeted to the active site of sterol 14α-demethylase, a cytochrome P450 protein encoded by the *CYP51* gene, which catalyzes demethylation of lanosterol in the ergosterol biosynthesis pathway. Differential sensitivity towards select azoles, such as the triazoles tetraconazole and propiconazole, has been demonstrated by in vitro comparisons between *Cps* and *Che*, where the latter species tolerated significantly higher exposure to the chemicals in a poison plate assay [3].

As with known antimicrobial compounds, repeated exposure to azoles places selective pressure on fungi, by which the *CYP51* target-site may gain resistance-conferring mutations that inhibit azole binding [11,12,13]. In addition to mutations in the active site, overexpression of the *CYP51* gene is also known to confer varying levels of resistance to azoles in diverse fungi [13,14]. Select lineages, including *Cps* and *Che*, are reported to possess three *CYP51* paralogs, denoted as *CYP51A*, *CYP51B*, and *CYP51C* [9,13,15,16]. From a comparative analysis of a worldwide collection of *Cps* and *Che* genomes, a *CYP51A* pseudogene was found in all examined *Cps* isolates from populations spanning four continents, but not in *Che* [16]. A premature stop codon occurring upstream of the translated active site, presumably disabling *CYP51A* function, was hypothesized to play a role in differential azole sensitivity seen in *Cps* relative to *Che* [16]. However, ergosterol biosynthesis is a multi-staged process involving over 20 genes, overexpression of which may serve as putative determinants of decreased azole sensitivity [17,18].

Transcriptomics studies have reported that when exposed to antifungals, fungi with reduced azole sensitivity display increased expression of genes from the xenome, a three-phase detoxification pathway found in fungi and other eukaryote lineages [19,20,21,22]. During the first phase, proteins oxidize harmful substances entering the cell [20]. In this context, cytochrome P450 monooxygenase enzymes aid metabolism of xenobiotics, such as antifungal drugs and plant secondary metabolites [20,21]. Although specific enzymes in this family can contribute to routine cellular activities/functions, such as lipid processing and synthesis of secondary metabolites, their role to aiding xenobiotic detoxification can be vital to non-target site mechanisms of tolerance to antifungals [21,22].

The second phase of detoxification involves deployment of proteins that perform chemical conjugation (modification) of xenobiotic substances [20]. Through conjugation the oxidized drug/toxin can be rendered more soluble, facilitating removal from the cell. Alternatively, the compound could be primed for breakdown or compartmentalized within the fungal cell. Among the proteins involved in this stage of detoxification are transferases, largely glutathione transferases [20,23,24]. The latter behaves by conjugating glutathione onto toxin molecules, facilitating efflux and thus reducing toxicity [23,24].

The final phase, known as efflux, is the removal of toxins, minimizing the opportunity for toxins to bind and inhibit cellular functions [13,20]. The ATP-binding cassette (ABC) transporters, ATP-dependent pumps that transport substances across membranes, are known to export toxins, including azoles, from the cell, and are often overexpressed in azole-resistant fungi [22,25,26]. Additionally, members of the major facilitator superfamily (MFS) of active transport proteins efflux azoles in parallel with ion movement along an electrochemical gradient [27,28]. Evidence in *Alternaria* has demonstrated that the loss of functional MFS transporters was linked with increased sensitivity towards fungicides, demonstrating the importance of efflux transporters in antifungal tolerance [28].

In light of previous findings on differential fungicide sensitivity in these two closely related sister species causing boxwood blight disease, we set out in the present study to examine gene expression patterns that may underpin their differential azole fungicide sensitivity [3,16]. We analyzed RNA-Seq expression data of control and tetraconazole-treated *Cps* and *Che* to examine *CYP51* paralog expression patterns, and to identify differentially over- and underexpressed genes in *Cps* and *Che* likely associated with xenobiotic detoxification or ergosterol biosynthesis [12,20,21]. Such genetic determinants could serve as potential targets for future functional studies and aid in teasing apart mechanisms of antifungal resistance to inform development and screening of novel antifungal compounds.

## 2. Results

### 2.1. Sensitivity of Cps and Che to Select Azole Fungicides

*Che* isolate CB45 had insignificantly higher EC_50_ and EC_85_ values when treated with either myclobutanil or tebuconazole (Table 1). *Cps* CT1 had an insignificantly higher EC_50_ value and an insignificantly lower EC_85_ value following treatment with triadimefon when compared to *Che* CB45 (0.86 times larger and ~1.41 times lower, respectively). However, *Che* isolate CB45 demonstrated substantially larger EC_50_ values when treated with tetraconazole (five times higher) or propiconazole (three times higher) compared with *Cps* CT1. CB45 also had a significantly larger EC_85_ value following tetraconazole treatment (over 16 times larger).

### 2.2. Quality Assessment of RNA-Seq Alignments

The number of CT1 control reads mapping to the CT1 genome ranged from 3,601,223 (~90.03%) to 3,773,581 (~94.34%), while the number of tetraconazole-treated reads mapping to the CT1 genome ranged from 3,589,726 (~89.74%) to 3,663,272 (~91.58%). For *Che*, the number of CB45 control reads mapping to the CB45 genome ranged from 3,747,123 (~93.68%) to 3,759,744 (~93.99%), while the number of tetraconazole-treated reads mapping to the CB45 genome ranged from 3,663,325 (~91.58%) to 3,767,407 (~94.19%).

### 2.3. Predicted Detoxification Genes in CT1 and CB45

CT1 and CB45 had 504 and 544 genes, respectively, possessing at least one of the four InterPro IDs associated with phases one and three of fungal xenobiotic detoxification (Tables S2 and S3) [20]. Using the broad GO name search, we identified 831 and 940 genes from CT1 and CB45, respectively, predicted to encode proteins with transferase activity (acetyl-CoA, glutathione, acetyl, etc.) (Tables S4 and S5).

### 2.4. Differentially Expressed Genes in Tetraconazole-Treated Cps

**Figure 1:**
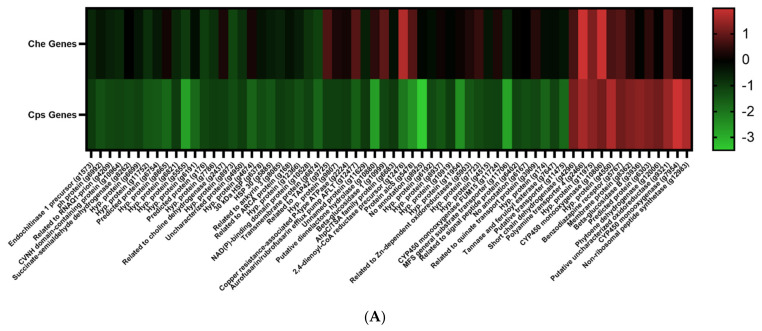

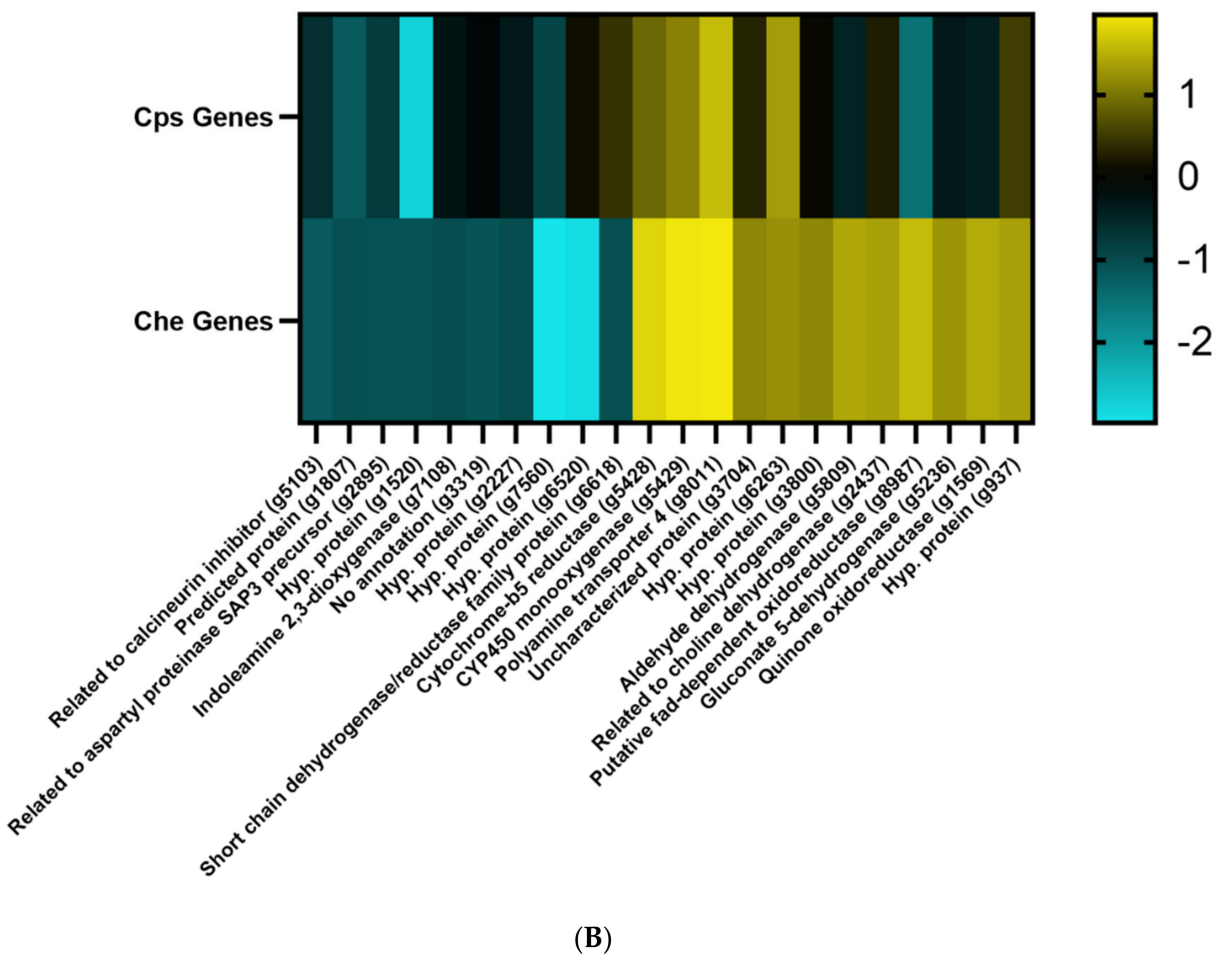
Heatmaps of significantly, differentially overexpressed and underexpressed genes in *Cps* and *Che* following tetraconazole treatment according to log_2_FC values. (**A**) Every gene significantly overexpressed or underexpressed in *Cps* is displayed along with the best corresponding multi-blast gene hit in *Che*. (**B**) All significantly expressed genes in *Che* are shown with their corresponding blastp hit in the *Cps* genome. In (**A**,**B**), we excluded genes from *Cps* or *Che* that did not possess any blastp hits within the other species’ genome. The genes are arranged according to increasing *p*-value (starting with the underexpressed genes). The name *hypothetical protein* was abbreviated to *Hyp. protein*. Heatmaps were generated from log_2_FC values using GraphPad Prism version 9.1.2 for Windows (GraphPad Software; San Diego, CA, USA, www.graphpad.com).

For all expression analyses, log_2_FC (fold change) values greater than 1.5 were considered overexpressed, as previously specified by [21], while log_2_FC values below −1.5 were considered underexpressed. Sixteen genes were statistically, significantly overexpressed in tetraconazole-treated CT1 compared to untreated CT1 (Table 2), and 55 genes whose expression significantly declined in CT1 following exposure to tetraconazole. Using our multi-blast analyses, we saw that 64 of these 71 total genes in *Cps* CT1 had a corresponding hit in *Che* CB45. None of the genes significantly overexpressed in CT1 were substantially underexpressed in CB45 (Table 2; Figure 1A). Rather, seven genes underexpressed in CT1 matched with overexpressed genes in CB45, though only one of which (a predicted 2,4-dienoyl-CoA reductase precursor, g12476) mapped to an overexpressed CB45 gene (log_2_FC > 1.5). Though generally not reaching our minimum threshold for a meaningful change in gene expression, genes in *Cps* CT1 following tetraconazole treatment were more likely to decline in expression.

**Figure 2:**
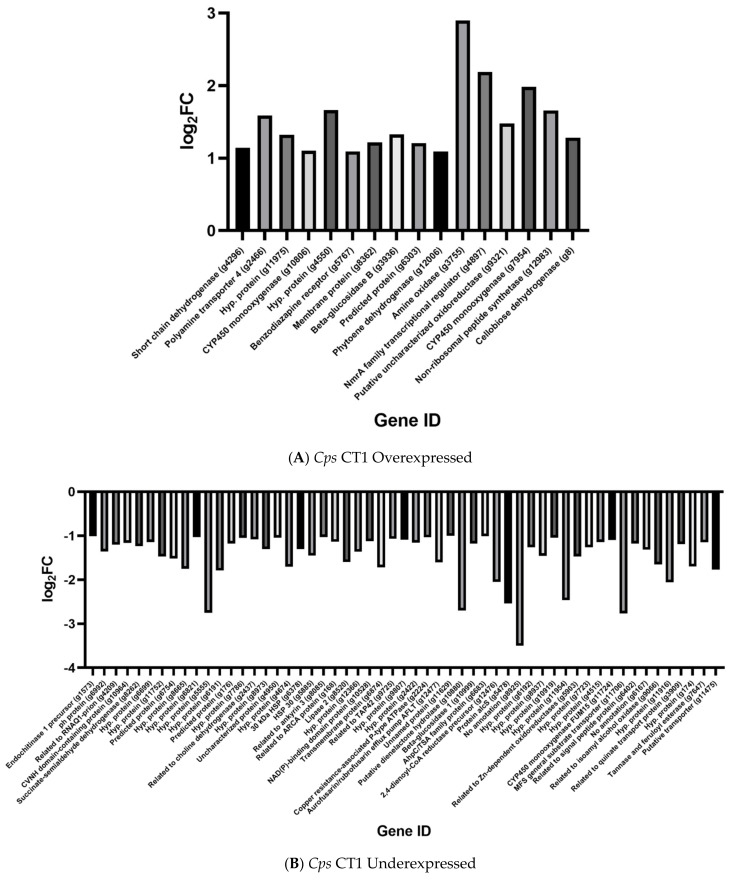

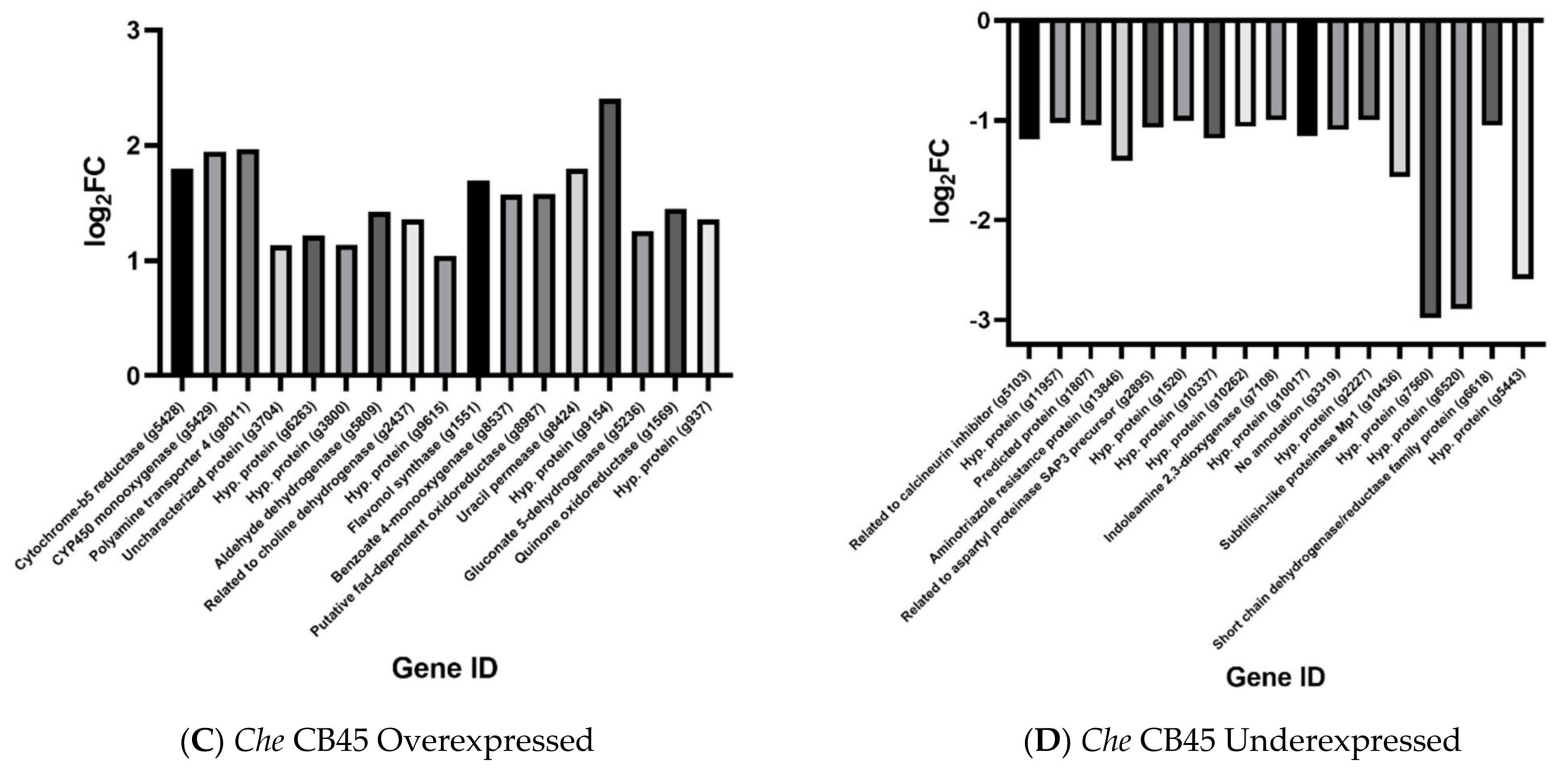
Bar charts of log_2_FC values of all differentially overexpressed and underexpressed genes in *Che* and *Cps* following tetraconazole treatment. Differentially expressed genes in *Cps* are displayed in (**A**,**B**), while differentially expressed genes in *Che* are shown in (**C**,**D**). *Hypothetical protein* was abbreviated to *Hyp. protein* for simplicity. Note that the *Cps* isolates had over double the number of differentially underexpressed genes than overexpressed genes, some of which were associated with efflux and detoxification activity. The genes are arranged according to increasing *p*-value. The data was plotted using GraphPad Prism version 9.1.2 for Windows (GraphPad Software; San Diego, CA, USA, www.graphpad.com).

We observed that only six of the genes had log_2_FC values greater than 1.5 (Table 2; Figure 2A). An amine oxidase gene was the most overexpressed among treated isolates (log_2_FC ≈ 2.90), though two of the genes were identified as encoding cytochrome P450 monooxygenases (one of which with a log_2_FC > 1.50), which can be putatively linked to chemical detoxification [20,21]. At least three genes were predicted to produce dehydrogenases (none with log_2_FC > 1.50), and one of the genes was predicted to yield a polyamine transporter protein (log_2_FC ≈ 1.59), which may furthermore be associated with detoxification [20]. There were 18 significantly underexpressed genes in CT1 whose log_2_FC values fell below -1.5 (Table 2; Figure 2B). Various proteins predicted to possess diverse functions declined in expression following azole treatment, such as a prion associated with epigenetic activity (g4209). However, three protein transporters exhibited large reductions in gene expression: a predicted efflux pump (g12477; log_2_FC ≈ −1.61), a putative transporter (g11475, log_2_FC ≈ −1.77), and a potential MFS transporter (g11706; log_2_FC ≈ −2.77).

**Figure 3:**
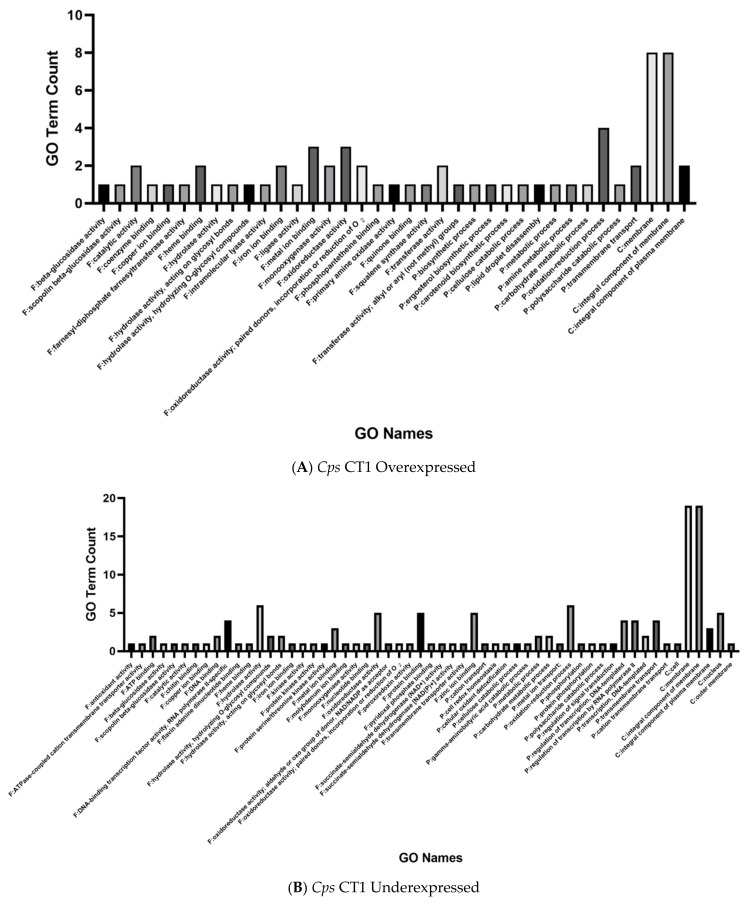

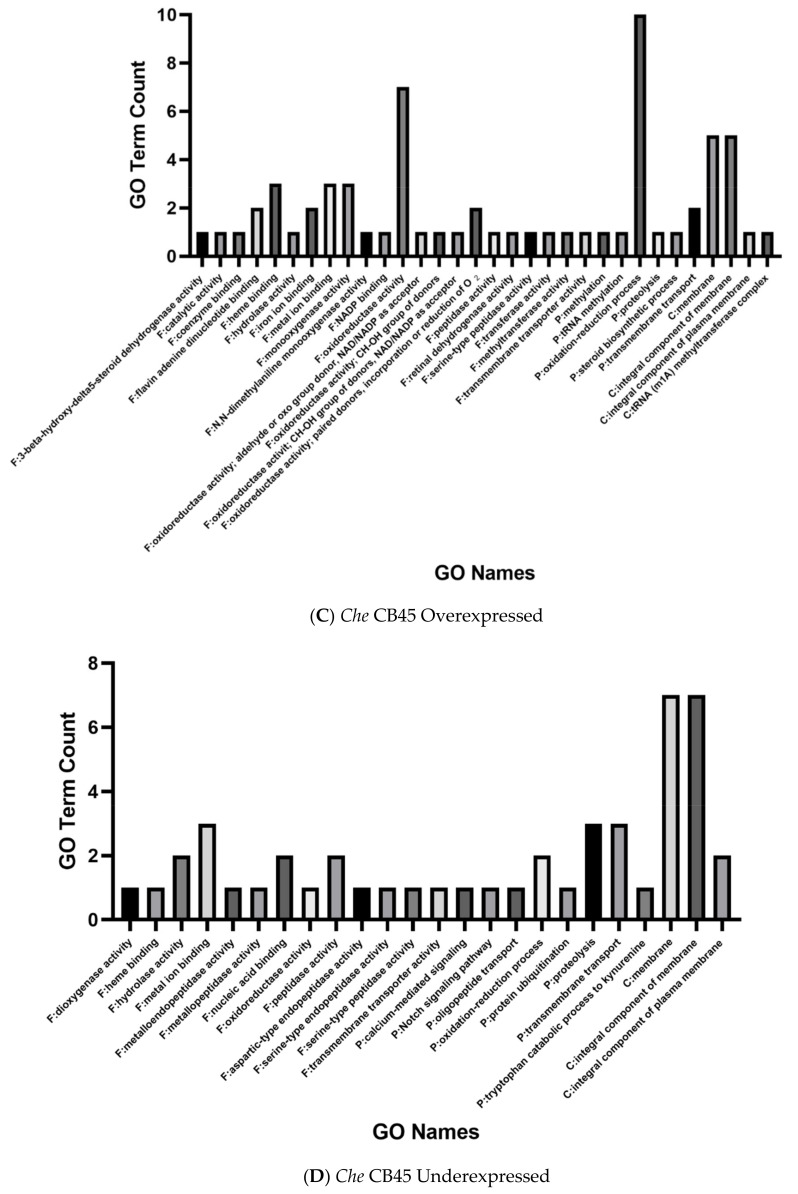
Bar charts displaying the total count for each GO annotation associated with significantly, differentially overexpressed and underexpressed genes in tetraconazole-treated *Cps* and *Che*. In (**A**,**B**), the counts are respectively shown for overexpressed and underexpressed genes in tetraconazole-treated *Cps*, while (**C**,**D**) show GO annotation counts for the overexpressed and underexpressed genes in *Che*. Note that *F:* refers to GO annotations describing *Molecular Functions*. A *Biological Process* is denoted by *P:*, and *Cellular Components* are denoted by *C:*. The data were plotted using GraphPad Prism version 9.1.2 for Windows (GraphPad Software; San Diego, CA, USA, www.graphpad.com).

The overexpressed genes in tetraconazole-treated *Cps* CT1 possessed a variety of different GO terms, though only the GO annotations associated with the membrane/integral component of the membrane appeared relatively common (occurring in eight of the 16 genes) (Figure 3A). Tetraconazole treatment appears to impact a large array of proteins in *Cps* CT1, such as proteins putatively associated with the nucleus (GO label occurring in five of 55 genes) or carrying out metal/zinc binding (occurring three and five times, respectively) and redox reactions (seen six times among 55 genes) (Figure 3B). Similar to the overexpressed genes, tetraconazole appears to significantly impact genes annotated to synthesize membrane proteins (observed in 19 of the 55 underexpressed genes).

### 2.5. Differentially Expressed Genes in Tetraconazole-Treated Che

Seventeen genes were statistically, differentially overexpressed in tetraconazole-treated CB45 compared to untreated CB45 (Table 3), and another 17 genes were significantly, differentially underexpressed in the tetraconazole-treated *Che* CB45. We observed that 22 of these 34 differentially over- and underexpressed genes successfully matched to a gene in *Cps* CT1 in our multi-blast analysis (Figure 1B). No gene underexpressed in tetraconazole-treated CB45 matched to a strongly overexpressed gene (log_2_FC > 1.5) in tetraconazole-treated CT1 (Figure 1B); however, seven of the overexpressed genes in *Che* did match *Cps* genes whose expressions were underexpressed (log_2_FC < −1.5) or unaffected (log_2_FC ≈ 0) following azole treatment (Figure 1B). In only one of these matches, the *Che* gene, the putative FAD-dependent oxidoreductase (g8987), had a log_2_FC exceeding 1.5, demonstrating that expression of this gene was particularly impacted by tetraconazole treatment in *Che* compared to the closest matching gene in *Cps*.

Eight of the overexpressed CB45 genes had log_2_FC > 1.50. The most overexpressed gene with a predicted function encoded a polyamine transporter 4 protein (log_2_FC ≈ 1.97), followed by a cytochrome P450 monooxygenase (log_2_FC ≈ 1.94) and a cytochrome-*b5* reductase (log_2_FC ≈ 1.80) (Table 3; Figure 2C). Several of these genes were thus annotated to have functions generally associated with xenobiotic detoxification activity [20]. Among the underexpressed genes in *Che*, four had log_2_FC values below −1.5, and only two (a predicted tyrosinase, g7560, according to our conserved domain search and a proteinase) had predicted function (Table 3; Figure 2D). Though possessing a log_2_FC value of roughly −1.4 and failing to reach our thresholds, there was a gene predicted to be associated with aminotriazole resistance that was underexpressed in *Che*.

When focusing on the GO annotations for these differentially over- and underexpressed genes, we noted that overexpressed genes in tetraconazole-treated CB45 tended to be associated with oxidoreductase activity (GO label occurred seven times among 17 genes) and redox processes (occurred in 10 of the 17 genes) (Figure 3C). A relatively high prevalence of overexpressed genes also tended to be linked to the cell membrane (occurred in five of 17 genes). For the underexpressed genes, the only GO names that prevalently re-occurred were those associated with the membrane or the integral component of the membrane (both being observed in seven of the 17 genes). Hence, tetraconazole exposure appeared to largely reduce expression of select genes associated with the cellular membrane.

### 2.6. CYP51A and CYP51B Expression Levels

In *Cps* isolate CT1, reads were mapped to the *CYP51A* pseudogene (g5325), though expression was low and statistically insignificant between the control and tetraconazole-treated cultures (CPM < 2; *p*-value > 0.05). The *CYP51B* gene (g5734) was considered significantly overexpressed in the treated culture based on *p*-value (<0.05), but the log_2_FC value failed to reach our threshold of 1.5 (~0.61).

Both the *CYP51A* (g2358) and *CYP51B* (g9283) genes were significantly overexpressed in the tetraconazole-treated, versus the untreated, *Che* CB45 culture (*CYP51A*: *p*-value ≈ 0.03; *CYP51B*: *p*-value << 0.05). However, in both instances, the log_2_FC values were less than 1.5 (*CYP51A*: ~0.63; *CYP51B*: ~0.46). The above data indicates that the expression patterns of the *CYP51* paralogs in *Cps* and *Che* were largely unaffected by tetraconazole treatment.

### 2.7. Prediction of Ergosterol Biosynthesis Genes

There were nine genes in CB45 predicted to play a role in ergosterol biosynthesis using our GO name search (Table 4). Two pairs of these genes were annotated to fulfill the same function (cytochrome P450 61; NADPH-cytochrome P450 reductase) and matched to the same genes in *Cps* according to our multi-blast analyses (g3336 and g9924, respectively). One of these genes, the predicted cytochrome P450 61 g133, had 100% identity to *Che* g1530 and *Cps* g3336 but was halted by a putative premature stop codon, upstream of predicted protein domains in the amino acid sequence of g1530. Although reads mapped to this gene, it is possible that *Che* g133 encodes a pseudogene unable to contribute to ergosterol biosynthesis, which may be complemented by g1530 in *Che*. Using data from [17] and NCBI blastp searches against *Fusarium* species, we identified an additional 17 predicted ergosterol biosynthesis genes in CB45 (Table 5). Notably, only one gene in the sensitive species, *Cps* g12006 (Table 4), showed significant overexpression in tetraconazole-treated CT1 (Table 1; Figure 1A, Figure 2A). No predicted ergosterol biosynthesis gene in CB45 show significant differences in gene expression.

## 3. Discussion

Boxwood blight is an economically important disease caused by two sister fungal species, *Cps* and *Che*, in landscape and plant nurseries across the globe [1,2]. As documented in a previous study [3] and reaffirmed in the analysis presented herein (Table 1), these two species differentially respond to select azole fungicides, the latter demonstrating a five-fold greater EC_50_ tolerance to tetraconazole, and significantly reduced sensitivity to propiconazole, both members of the azole class (Table 1). In this study, we applied transcriptomic and bioinformatic analyses to gain insight into possible genetic determinants for the observed differences in azole fungicide sensitivity.

Results from our previous study showed that a *CYP51A* pseudogene in *Cps* may explain differential sensitivity to azoles [16]. As expected, few reads and no statistically significant difference in gene expression were detected for the *CYP51A* pseudogene in *Cps*. Given the stringent mapping parameters applied here, and the observation that the few reads that were mapped to the *CYP51A* pseudogene mapped to the region upstream of the premature stop codon, we can assume that a lack of functional CYP51A protein expression could result in less compensatory ergosterol synthesis. By contrast, in *Che*, the expression of both *CYP51A* and *CYP51B* gene paralogs could result in greater ergosterol synthesis, as both CYP51A and CYP51B proteins are known to catalyze the same step in the ergosterol pathway. In *Candida albicans*, chromosomal duplications of *CYP51* are linked to fluconazole-resistance [29]. Numerous studies demonstrate overexpression of *CYP51* as a mechanism for resistance to azoles (reviewed in [30]). Future complementation studies with a functional *CYP51A* in *Cps* are warranted to carefully assess the impact of the *CYP51A* pseudogene.

Although *CYP51* and its paralogs receive the majority of focus when it comes to azole fungicide resistance, the synthesis of ergosterol is a complex metabolic process driven by over 20 genes from as many as three separate biochemical pathways [17,18]. Here we identified potential ergosterol biosynthesis genes in *Cps* and *Che*, though, intriguingly, only one biosynthesis gene, observed only in our sensitive *Cps* isolate (g12006, Table 2 and Table 4), showed significant overexpression following one hour after treatment. This finding suggests that *Che* is not reliant upon upregulation of ergosterol biosynthesis following exposure to offset the inhibitory effects of tetraconazole. Interestingly, our observations differ from those observed by [31] in *Cochliobolus sativus*. This pathogen, which exhibits emerging fungicide resistance, displayed gene expression patterns whereby select ergosterol biosynthesis pathway genes were upregulated within hours of exposure. Our results in *Che* may instead support the hypothesis that the presence of an additional, functional *CYP51* paralog in *Che* could confer the increased tolerance towards azoles relative to *Cps* [16]. In *Schizosaccharomyces pombe*, it was demonstrated that the addition of a gene encoding a C-5 sterol desaturase participating in ergosterol biosynthesis increased synthesis of ergosterol and was correlated with improved tolerance towards exposure to extreme temperatures and the presence of ethanol [32]. In light of the differential thermotolerance previously reported in *Cps* and *Che*, comparative analyses of lipid profiles and ergosterol levels are warranted in these two species [3].

Analysis of RNA-Seq expression profiles of tetraconazole-treated *Cps* and *Che* revealed overexpressed genes predominantly associated with oxidoreductase activity, oxidation-reduction processes, and monooxygenase activity, somewhat parallel to findings from previous transcriptomic studies of azole sensitivity in fungi (Table 3 and Table 4) [17,21,31]. The overexpression of cytochrome P450 monooxygenases is implicated in phase one of xenobiotic detoxification, two of which have been functionally shown to play a role in multidrug resistance in the turfgrass fungal pathogen, *Clarireedia jacksonii* (synonym = *Sclerotinia homoeocarpa*) [20,21,33]. Furthermore, our candidate detoxification genes and their differential expression patterns in *Che* and *Cps* were consistent with findings from previous studies in both *Cercospora beticola* and *Clarireedia jacksonii* [17,21]. The putative detoxification genes identified in the current study, including subsets of predicted transferases (Tables S5 and S6), could provide a more comprehensive list of putative targets for functional characterization and/or serve as genetic markers for monitoring the evolution of resistance and potentially guiding future fungicide development. It should be noted that gene deletion experiments in *Cercospora beticola* to verify the role of candidate detoxification genes were inconclusive, while in *Clarireedia jacksonii* experiments validated the function of two novel cytochrome P450 monooxygenases [17,21]. The amenability to genetic transformation of *Cps* and *Che* remains unknown, but findings presented here serve to build a knowledge base to enable such studies.

As reported by [21], non-synonymous polymorphism in a zinc transcription factor led to gain-of-function resulting in overexpression of detoxification genes from the aforementioned gene families of the fungal xenome. The direct comparisons of gene expression patterns relied on the apparent clonal nature of the sensitive and resistant fungal isolates of *Clarireedia jacksonii*, which also facilitated screening for transcription factor polymorphism. However, in the current study, the genetic divergence and the higher levels of genetic polymorphism between *Cps* and *Che* (warranting their placement in two separate species [3]) hampered efforts to readily identify polymorphism that could be linked to differential expression of an assortment of genes representing multiple gene families. It should be noted that a homolog of a negative regulator of nitrate metabolism genes, *nmrA* (g4897), was underexpressed in tetraconazole-treated *Cps* (Table 2; Figure 1A, Figure 2B). Though the linkage of this gene to azole sensitivity has not been previously reported, *nmrA* deletion mutants have been shown to display reduced sensitivity to rapamycin and methylmethanesulfonate, presumably through rapamycin signaling and DNA-damage [34,35]. Another candidate gene from *Cps* that could serve to influence expression levels of downregulated genes shows similarity to RNQ1 (Table 2; Figure 1A, Figure 2B), a prion encoding gene that is thought to have a role in epigenetics and phenotypic changes in *Saccharomyces cerevisiae*, and which also mediates polyQ aggregation in concert with heat shock proteins in a Huntington’s Disease yeast model [36,37]. The significance of these genes in the response to an inhibitory concentration of tetraconazole by *Cps* remains to be determined.

Numerous genes were downregulated in *Cps* in response to tetraconazole treatment (Table 2; Figure 1A, Figure 2B), which may reflect the shunting of energy from housekeeping and maintenance to xenobiotic detoxification, analogous to findings from [21], which found a several-fold greater abundance of genes downregulated in the sensitive isolates following exposure to azole exposure, including genes encoding proteins with predicted association with cell membranes. In both [21] and the current study, the sensitive isolate/species were exposed to an azole concentration of five times the EC_50_ or above, presumably a lethal dose, which could explain the much greater number of downregulated genes and the fact that equivalent genes in *Che* tended to be unaffected/upregulated (Figure 1A). Further, several of these genes were predicted to encode efflux transporter proteins (Table 2; Figure 1A, Figure 2B) [13,20,25,26,27,28]. Given the sensitivity of *Cps* CT1 to tetraconazole and its aforementioned exposure to an apparent lethal dosage of the chemical, fungal cells may have in response triggered reduction in expression of suites of genes unrelated to azole detoxification.

In *Che*, several overexpressed genes are associated with the first and third phases of detoxification, with GO terms suggesting association with the cell membrane and oxidoreductase activity (Table 3; Figure 1B, Figure 2C, Figure 3C). A similar pattern is reported in *Cochliobolus sativus*, *Cercospora beticola* and *Clarireedia jacksonii* which, following azole treatment, display significant increases in gene expression for phase I cytochrome P450 proteins and phase III ABC-G transporters [17,21,31]. Of particular interest is a cytochrome P450 gene described as a benzoate-4-monooxygenase, which showed significant overexpression (g8537). A previous study on antifungal response in *Fusarium oxysporum* and *Neocosmospora solani* found a benzoate-4-monooxygenase to be overexpressed following treatment with amphotericin b and posoconazole [38]. This gene, denoted *CYP53*, is a member of the family of CYP450 enzymes exclusive to fungi that are involved in phenolic detoxification. A study looking into natural compounds to target *Cochliobolus lunatus* found that natural phenolic compounds inhibiting *CYP53A15* activity showed antifungal activity [39]. Perhaps this gene target may serve to inform novel fungicide treatments in boxwood blight.

Few genes were underexpressed in *Che* following treatment, and, of note, included several genes with predicted peptidase activity (Table 3; Figure 1B, Figure 2D). Aspartyl peptidase activity has been shown to vary in response to azoles in a *Candida albicans*, though future studies are necessary to begin to elucidate how peptidase activity can influence azole sensitivity in fungi [40]. According to our conserved domain search, a predicted tyrosinase gene (g7560) showed the highest degree of underexpression following tetraconazole exposure, and though azole chemicals are known inhibitors of tyrosinase activity, their impact on gene expression remains unclear [41]. A third gene of interest, a predicted short-chain dehydrogenase, showed a minimal degree of underexpression following tetraconazole exposure. In *Aspergillus fumigatus*, an important human pathogen, deletion mutants for *HorA* (a mitochondrial short-chain dehydrogenase) showed increased tolerance to azoles, suggesting to be indicative of a metabolic state linked to mitochondrial impairment [42]. Taken together, diverse metabolic pathways appear to be downregulated in *Che* to facilitate azole tolerance.

## 4. Materials and Methods

### 4.1. Estimating Effective Fungicide Concentrations for 50% and 85% Inhibition of Growth In Vitro of Cps and Che

To identify fungicides to which *Cps* and *Che* are differentially tolerant, in vitro radial-growth assays were used with a range of concentrations of fungicides, as listed in Table S1. All assays were performed using *Cps* specimen CpsCT1s (hereafter: CT1) and *Che* type specimen CB045 (hereafter: CB45); for tetraconazole and propiconazole, an additional isolate of each species was used, as indicated in Table S1. All assays used commercial formulations of fungicides (Table S1), added to autoclaved half-strength potato-dextrose agar (half-PDA) in amounts producing the concentration ranges specified in Table S1, based on labeled percentages of active ingredient. After thorough mixing, exactly 20 mL of the amended media (or unamended control media) were then poured into 100 × 15 mm plastic Petri dishes, which were then used the following day for inoculation with test isolates. Inoculations of amended media used 10-day-old cultures growing on Spezieller Nährstoffarmer agar (SNA) [43], using 5.0-cm diameter plugs taken with a cork borer from culture margins and placed in the center of the dish. Two replicates were used for each isolate x treatment/control combination. After sealing plates with Parafilm M^®^ (Amcor, Zurich, Switzerland), cultures were incubated at room temperature in the dark until control cultures (i.e., those not amended with fungicide) reached no less than 1.0 cm from the dish perimeter, for the number of days as indicated in Table S1. Probit analysis was used to determine the effective concentrations of fungicide necessary for 50% and 85% inhibition of growth, as follows. Diameters were measured using digital calipers (Mahr GmbH, Esslingen am Neckar, Germany), with measurements automatically uploaded into a spreadsheet. After subtracting the inoculation-plug diameter, growth rates were calculated by dividing adjusted diameters by the number of growth days, then normalized by dividing by the growth rate of the unamended control. These normalized growth rates, expressed as percentages, were then transformed to “probits” (probability units) [44]. The linearized regression of probits against log-transformed fungicide concentrations was then used to calculate EC_50_ and EC_85_ values, as listed in Table S1.

### 4.2. Genome Sequencing and Assembly

The genome sequences and predicted genes of *Cps* isolate CT1 (also known as isolate CBS 139,707 and cpsCT1) and *Che* isolate CB45 (also known as CBS 138,102 and CB045) reported in [16] were used to guide our differential expression analysis.

### 4.3. RNA Sequencing of Control versus Tetraconazole-Treated CT1 and CB45

Using 10 mL of half-strength PDB, conidia were washed from ten-day-old cultures of CT1 and CB45, growing on half-strength PDA that had been scored four days previously with a sterile scalpel to promote sporulation. After estimating conidia concentrations with a hemocytometer, 1000 conidia were plated onto half-strength PDA and measured the following day for viability. Based on those estimates, 10,000 putatively viable conidia were used to inoculate each of the sixteen 50-mL tubes containing 20-mL of half-strength PDB, placed at a 45° angle to maximize surface area, and allowed to grow at room temperature without shaking for six days. The resulting mycelial mats were then transferred with sterile spatulas to sixteen 50-mL tubes containing 20 mL of half-strength PDB, half of which had been amended with 1.0 ppm tetraconazole (Minerva). After incubation on a rocking platform for one hour, the mycelial mats were blotted on Kimwipes over ice, then transferred to 2-mL tubes, plunged in liquid N_2_, and lyophilized. The resulting lyophilized mycelium was stored at −80 °C for one day before RNA extraction using the Ambion Trizol Plus kit (ThermoFisher Scientific, Waltham, MA, USA). Resulting RNAs were then processed at the Yale Center for Genome Analysis (West Haven, CT, USA), where they were first analyzed for quality and quantity on a Bioanalyzer (Agilent Technologies, Inc., Santa Clara, CA, USA), confirmed to have RIN scores of 9.0 or greater, and then processed for 2 × 75-bp next-generation sequencing on an Illumina HiSeq-2500 (Illumina, Inc., San Diego, CA, USA).

### 4.4. Genome Functional Annotation and Multi-Blast

Unless specified otherwise, the following procedures were performed using OmicsBox software [45]. For both *Calonectria* genomes, the protein sequences of each predicted gene were extracted using our gene annotation files. A blastp-fast search was performed in September 2019 for each gene using the OmicsBox CloudBlast feature and the nr_v5 non-redundant protein sequence database using the following parameters: searches limited to fungi (taxa: 4751); *e*-value ≤ 10^−10^; word size = 6; retaining only the top 10 blast hits for each amino acid sequence. A blast description annotator and a filter removing matches with regions of low complexity were applied. After performing the CloudBlast search, an InterProScan analysis (CloudIPS) acquired protein domain information for each protein in our files [46]. All available databases other than PatternScan, Coils, and MobiDBLite were selected for this analysis. Finally, Gene Ontology (GO) mapping was performed on each protein sequence in the ensuing file using the most recently updated GO database available [46].

The amino acid sequences for predicted genes in CB45 and CT1 were extracted using OmicsBox [45]. Two multi-blasts (blastp) were performed using the CLC Genomics Workbench 11.0 (QIAGEN, 2017), the first querying CB45 genes against CT1 and the second querying CT1 genes against those of CB45. The same parameters were used for both BLAST searches (match = 2, mismatch = -3; gap existence cost = 5, extension cost = 2; *e*-value ≤ 10^−10^; word size = 11; number of threads = 10; filter low complexity matches). The two multi-blasts allowed for verification of whether we could find reliable matches of equivalent CB45 genes in the CT1 genome.

### 4.5. Identification of Candidate Detoxification Genes

Genes with protein domains associated with the three phases of detoxification (oxidation, conjugation, and efflux) were mined from the CT1 and CB45 annotation files [20]. Amino acid sequences possessing any of the following four InterPro IDs were extracted from the annotation files for both species: Cytochrome P450 (IPR001128; oxidation/breakdown phase); Major Facilitator Superfamily (IPR020846; MFS, efflux phase); PDR-like Subfamily G, Domain 1 (IPR034001; ABC-transporter, efflux phase); and PDR-like Subfamily G, Domain 2 (IPR034003; ABC-transporter, efflux phase). Transferases were extracted by filtering the GO-annotated CT1 and CB45 genomes for the GO function name *transferase activity*. The full lists of these genes are stored as Supplementary Tables S3–S6.

### 4.6. RNA-Seq Analysis of Control versus Tetraconazole-Treated Isolates

Three biological replicates were sequenced for control and tetraconazole-treated CT1 and CB45. Each biological replicate had three technical replicates. In total, there were 36 experimental units, each with a minimum of 4,000,000 total RNA-Seq reads.

All of the following procedures in this section were performed using the OmicsBox software [45]. RNA-Seq reads were converted into FASTQ file format and read file quality was assessed and poor reads were filtered out using the default parameters of FastQC version 0.11.8 [47]. RNA-Seq reads meeting the quality threshold for CT1 and CB45 isolates were, respectively, aligned to the CT1 and CB45 reference genomes using the STAR aligner software in the OmicsBox environment [48], with read overhang set to one base pair lower than the maximum read length (75 base pairs) following the manufacturer’s protocol. The annotation files of the two genomes were used to help guide the alignment. The assigned minimum and maximum intron lengths were 20 base pairs and 2000 base pairs, respectively [49]. Only one mismatch between the RNA-Seq read and the reference genome was permitted, and reads were allowed to match with only one region in the genome. These stringent mapping parameters were used to diminish the likelihood of false matching between RNA reads and predicted genes.

Using the HTSeq 0.9.0 package, two read count tables were generated, one comparing control and treated CT1 RNA-Seq reads and the other comparing control and treated CB45 reads [50]. The CT1 and CB45 structural annotation files were respectively used to acquire the RNA-Seq read counts per gene. The counts were made according to read overlap with the exon region of each gene (overlap mode: union; strand specificity: non strand specific). Since Illumina RNA-Seq procedures generate high-quality reads with low error rates (less than one base in 500), a high minimum mapping quality value of 25 (or an error rate of one in 500 bases) was specified for this step.

Using the edgeR software package within the OmicsBox environment and our count tables, separate differential expression analyses were run for CT1 and CB45 [51]. In both instances, a count per million (CPM) filter was set to one and at least one sample needed to reach this criterion. These parameters indicated that, for every million reads in a technical replicate, at least one of the reads must map to a particular gene for that gene to be included in the analysis. On average, there were between three and four million reads which mapped to a reference genome per replicate. Thus, for a given gene to be included in this analysis, at least three reads were needed to map to that gene for a minimum of one replicate. Since there were many genes whose samples possessed two or fewer matches, this criterion was considered suitable to acquire reliable calculations of gene differential expression. Additionally, the strict RNA-Seq alignment conditions decreased the likelihood that any of the 75-bp reads were mapped to similar regions of incorrect genes, thereby increasing confidence that only genes with reliably mapped reads were included in this analysis. However, we had also run each differentially over- and underexpressed gene against the NCBI Conserved Domain Search tool to further check the predicted functions of these genes. Non-matching genes were described as encoding a hypothetical protein. GraphPad Prism version 9.1.2 for Windows (GraphPad Software; San Diego, CA, USA, www.graphpad.com) was used to generate plots displaying normalized gene expression values and GO annotation counts for the differentially expressed genes in *Cps* CT1 and *Che* CB45 treated with tetraconazole.

### 4.7. Identification of Ergosterol Biosynthesis Genes

A list of putative ergosterol biosynthesis genes were extracted from our annotated *Che* CB45 genome using the GO name *ergosterol biosynthetic process*. A protein blast was also performed against the CB45 genome (gap existence cost = 11; gap extension cost = 1; *e*-value = 10^−10^, word size = 3; number of threads = 4; filter low complexity matches; BLOSUM62) using various NCBI reference ergosterol biosynthesis genes from *Fusarium* species (Table S6). This step was performed to try and capture putative ergosterol biosynthesis genes missing from our GO name search. We used the pathway discussed by [17] to help us guide our NCBI gene search. Using the two aforementioned multi-blasts (see *Genome functional annotation and multi-blast*), we documented the closest matching genes in *Cps* CT1.

## 5. Conclusions

The findings presented here contribute to the body of knowledge on azole resistance determinants in filamentous fungal plant pathogens, and though previous transcriptomics studies of this nature have undertaken similar methodologies, the novelty of this study lies in part in the phylogenetic relationship of *Cps* and *Che*, two distinct sister species, and the presence of a *CYP51A* pseudogene in *Cps*. Follow up studies may have relevance to novel azole resistance mechanisms in human fungal pathogens [6]. Many medically important fungal pathogens, including *Aspergillus fumigatus* and *Candida albicans*, have shown reduced sensitivity and/or resistance towards azole antifungals. *Candida auris*, a species formally described in 2009 [52,53] is comprised of distinct clades, and treatment for disease from this pathogen is complicated by azole resistance and resistance to other major groups of medical antifungals through target gene mutations, gene duplications, and increased expression of efflux proteins. As new reports of antifungal resistance continue to emerge, an increasing need for novel drug targets arises. Future transcriptomic studies of this nature will provide a roadmap to facilitate development of improved fungicides and antifungal drugs for medicine, while serving to uncover cross-species patterns and novel mechanisms underpinning multidrug resistance in fungi.

## Figures and Tables

**Table 1:** Effective concentrations of fungicides restrict *Cps* and *Che* growth.

Fungicide	EC_50_, ppm		EC_85_, ppm	
	*Cps*	*Che*	*Cps*	*Che*
Myclobutanil	0.43	0.57	3.10	4.60
Tetraconazole *	**0.20**	**1.00**	**0.60**	**9.70**
Tebuconazole	0.10	0.13	0.56	0.86
Triadimefon	5.00	4.30	26.20	37.00
**Propiconazole**	**0.05**	**0.15**	0.56	0.84

* Values in bolded text show significant difference in EC_50_ and EC_85_ values.

**Table 2:** Significantly, differentially overexpressed genes in tetraconazole-treated *Cps* CT1 when compared to untreated CT1.

Name	Description (Blastp Result)	*e*-Value	Mean Similarity (%)	Fold Change	log_2_FC *	*p*-Value
g3755	Amine oxidase	0	82.66	7.44	2.90	0.0020
g4897	NmrA family transcriptional regulator	2.86E-171	70.67	4.56	2.19	0.0021
g7954	Cytochrome P450 monooxygenase	3.94E-173	87.1	3.95	1.98	0.0043
g4550	Hypothetical protein AK830_g2355	9.20E-115	74.11	3.17	1.66	5.63 × 10^−8^
g12983	Non-ribosomal peptide synthetase	0	64.5	3.15	1.66	0.0044
g2466	Polyamine transporter 4	0	82.16	3.01	1.59	1.97 × 10^−13^
g9321	Putative uncharacterized oxidoreductase	1.53E-124	71.69	2.78	1.48	0.0041
g3936	Beta-glucosidase B	0	80.2	2.51	1.33	2.68 × 10^−4^
g11975	Hypothetical protein CEP53_001993	2.59E-98	82.1	2.50	1.32	1.47 × 10^−11^
g8	Cellobiose dehydrogenase	1.32E-167	70.38	2.43	1.28	0.0061
g8362	Membrane protein	9.24E-60	72.66	2.33	1.22	3.54 × 10^−5^
g6303	Predicted protein	1.37E-21	71.72	2.31	1.21	3.81 × 10^−4^
g4296	Short chain dehydrogenase	4.53E-142	86.45	2.21	1.14	4.66 × 10^−17^
g10806	Cytochrome P450 monooxygenase	0	93.45	2.15	1.10	1.00 × 10^−9^
g5767	Benzodiazapine receptor	7.02E-110	88.73	2.13	1.09	7.92 × 10^−8^
12006	Phytoene dehydrogenase	0	76.86	2.13	1.09	4.90 × 10^−4^
g11629	Unnamed protein product	3.81E-83	51.01	−2.00	−1.00	0.0015
g6683	AhpC/TSA family protein	3.46E-121	88.47	−2.01	−1.01	0.0033
g1573	Endochitinase 1 precursor	0	82.40	−2.01	−1.01	6.65 × 10^−73^
g8085	Related to ankyrin 3	1.03E-144	52.21	−2.04	−1.03	2.05 × 10^−6^
g6821	Hypothetical protein AK830_g533	0	82.08	−2.04	−1.03	1.01 × 10^−22^
g2224	Copper resistance-associated P-type ATPase	1.42E-93	64.75	−2.05	−1.04	0.0011
g10919	Hypothetical protein FOYG_12748	6.12E-44	46.12	−2.06	−1.04	0.0089
g4950	Uncharacterized protein FVRRES_10874	1.61E-115	55.96	−2.06	−1.04	4.86 × 10^−9^
g7786	Hypothetical protein PEX2_036530	7.21E-71	67.85	−2.07	−1.05	3.91 × 10^−11^
g9725	Related to TAP42, component of the Tor signaling pathway	0	87.44	−2.10	−1.07	1.38 × 10^−4^
g2437	Hypothetical protein AK830_g6151	2.77E-115	52.64	−2.12	−1.09	9.04 × 10^−11^
g9807	Hypothetical protein	0	71.60	−2.13	−1.09	2.27 × 10^−4^
g11724	Cytochrome P450 monooxygenase FUM15	0	93.17	−2.14	−1.10	0.0173
g10528	NAD(P)-binding domain protein	2.54E-118	76.44	−2.18	−1.13	4.35 × 10^−5^
g168	Related to ARCA protein	0	69.08	−2.20	−1.14	2.12 × 10^−6^
g6699	Hypothetical protein AK830_g11121	0	79.89	−2.21	−1.14	4.70 × 10^−40^
g4515	Hypothetical protein FCULG_00008984	0	72.15	−2.21	−1.14	0.0149
g7647	Tannase and feruloyl esterase	0	74.16	−2.22	−1.15	0.0463
g2422	Hypothetical protein S40288_06769	3.29E-96	62.25	−2.23	−1.16	3.62 × 10^−4^
g10964	CVNH domain-containing protein	1.00E-76	64.11	−2.24	−1.16	1.13 × 10^−47^
g10999	Beta-glucosidase 1	0	76.92	−2.26	−1.17	0.0027
g6402	Related to signal peptide protein	4.11E-90	60.69	−2.26	−1.18	0.0202
g176	Predicted protein	3.17E-42	54.15	−2.26	−1.18	8.24 × 10^−12^
g3909	Related to quinate transport protein	0	91.93	−2.29	−1.19	0.0283
g4209	Related to RNQ1-prion, epigenetic modifier of protein function	1.29E-129	67.60	−2.30	−1.20	3.31 × 10^−52^
g8262	Succinate-semialdehyde dehydrogenase	0	91.28	−2.36	−1.24	4.45 × 10^−45^
g6192	Hypothetical protein CSUB01_04341	9.35E-59	57.37	−2.40	−1.26	0.0077
g7723	Hypothetical protein CEP52_010997	6.25E-114	63.88	−2.40	−1.27	0.012
g6378	30 kDa heat shock protein	1.64E-100	80.62	−2.47	−1.30	3.92 × 10^−7^
g8973	Hypothetical protein BFJ68_g11167	0	54.02	−2.47	−1.30	8.85 × 10^−10^
g6167	No successful annotation	--	--	−2.49	−1.32	0.0227
g6992	ph protein	4.28E-156	82.60	−2.56	−1.36	2.05 × 10^−61^
g12366	Hypothetical protein	9.53E-118	67.61	−2.58	−1.37	1.07 × 10^−5^
g5885	Heat shock protein 30	1.21E-107	79.46	−2.73	−1.45	1.24 × 10^−6^
g8937	Hypothetical protein CDD83_2148	1.87E-71	62.41	−2.74	−1.45	0.0087
g11752	Hypothetical protein	152E-57	65.38	−2.77	−1.47	1.58 × 10^−35^
g5903	Related to Zn-dependent oxidoreductases	0	85.94	−2.78	−1.47	0.0122
g6754	Predicted protein	0	83.81	−2.86	−1.52	7.78 × 10^−28^
g8520	Lipase 2	0	63.85	−3.02	−1.59	4.58 × 10^−6^
g12477	Aurofusarin/rubrofusarin efflux pump AFLT	0	91.34	−3.04	−1.61	0.0015
g9066	Related to isoamyl alcohol oxidase	0	77.69	−3.15	−1.65	0.0243
g174	Hypothetical protein DV735_g44, partial	3.18E-51	46.99	−3.25	−1.70	0.0289
g4674	Hypothetical protein CEP53_004141	5.28E-65	53.38	−3.26	−1.71	6.35 × 10^−8^
g6874	Transmembrane protein	2.43E-107	79.26	−3.29	−1.72	1.13 × 10^−4^
g8665	Hypothetical protein AK830_g8487	1.99E-82	60.83	−3.36	−1.75	2.49 × 10^−25^
g11475	Putative transporter	0	86.43	−3.41	−1.77	0.0468
g6191	Hypothetical protein CNYM01_02327	1.69E-176	55.57	−3.46	−1.79	7.71 × 10^−13^
g12476	2,4-dienoyl-CoA reductase precursor	8.00E-175	76.95	−4.14	−2.05	0.0038
g11916	Hypothetical protein CSHISOI_01161	2.78E-33	44.31	−4.16	−2.06	0.0282
g11954	Hypothetical protein AK830_g7182	6.58E-111	71.82	−5.51	−2.46	0.0097
g5478	Protein alcS	6.31E-101	77.15	−5.81	−2.54	0.0060
g10880	Putative dienelactone hydrolase protein	1.73E-150	80.87	−6.49	−2.70	0.0025
g5555	Hypothetical protein AK830_g4498	8.40E-104	80.07	−6.72	−2.75	5.88 × 10^−19^
g11706	MFS general substrate transporter	4.10E-128	70.32	−6.80	−2.77	0.0183
g8925	No successful annotation	--	--	−11.32	−3.50	0.0074

* The table is arranged from most to least differentially overexpressed gene (highest fold change and log_2_FC to least).

**Table 3:** Significantly, differentially overexpressed genes in tetraconazole-treated *Che* CB45 when compared to untreated CB45.

Name	Description (Blastp Result)	*e*-Value	Mean Similarity (%)	Fold Change	log_2_FC *	*p*-Value
g9154	Hypothetical protein CEP51_000673	2.20E-132	76.62	5.29	2.40	0.0025
g8011	Polyamine transporter 4	0	81.96	3.91	1.97	2.31 × 10^−25^
g5429	Cytochrome P450 monooxygenase	0	93.05	3.85	1.94	5.11 × 10^−34^
g8424	Uracil permease	0	75.19	3.48	1.80	0.0023
g5428	Cytochrome-b5 reductase	0	84.22	3.48	1.80	3.68 × 10^−39^
g1551	Flavonol synthase	0	89.89	3.23	1.69	0.0013
g8987	Putative fad -dependent oxidoreductase protein	0	82.94	2.99	1.58	0.0018
g8537	Benzoate 4-monooxygenase	0	89.37	2.97	1.57	0.0015
g1569	Quinone oxidoreductase	0	78.15	2.73	1.45	0.0034
g5809	Aldehyde dehydrogenase	0	80.98	2.68	1.42	2.43 × 10^−4^
g2437	Related to choline dehydrogenase	0	85.31	2.56	1.36	6.02 × 10^−4^
g937	Hypothetical protein	0	79.28	2.56	1.36	0.0038
g5236	Gluconate 5-dehydrogenase	0	95.34	2.39	1.26	0.0027
g6263	Hypothetical protein CEP53_001993	2.24E-100	82.91	2.32	1.22	4.46 × 10^−11^
g3800	Hypothetical protein NECHADRAFT_97613	3.70E-162	76.9	2.20	1.14	2.14 × 10^−9^
g3704	Uncharacterized protein FFFS_03043	0	64.92	2.20	1.13	1.43 × 10^−16^
g9615	Hypothetical protein CEP53_001289	3.14E-132	79.07	2.06	1.04	8.46 × 10^−4^
g7108	Indoleamine 2,3-dioxygenase	0	84.17	−2.00	−1.00	2.72 × 10^−7^
g2227	Hypothetical protein AK830_g4468	1.43E-24	58.42	−2.00	−1.00	1.21 × 10^−4^
g1520	Hypothetical protein AK830_g4498	6.07E-105	80.02	−2.01	−1.01	1.24 × 10^−18^
g11957	Hypothetical protein M419DRAFT_5450	3.76E-89	52.21	−2.03	−1.02	2.57 × 10^−44^
g1807	Predicted protein	2.77E-42	65.74	−2.07	−1.05	1.34 × 10^−30^
g6618	Short chain dehydrogenase/reductase family protein	1.21E-113	70.07	−2.07	−1.05	0.0016
g10262	Hypothetical protein AK830_g1682	1.79E-33	54.49	−2.09	−1.06	6.10 × 10^−9^
g2895	Related to aspartyl proteinase SAP3 precursor	0	71.46	−2.11	−1.07	2.77 × 10^−22^
g3319	No successful annotation	--	--	−2.14	−1.10	1.80 × 10^−6^
g10017	Hypothetical protein AK830_g9553	2.46E-77	57.73	−2.23	−1.16	9.33 × 10^−7^
g10337	Hypothetical protein FOTG_02367	0	53.82	−2.27	−1.19	4.28 × 10^−18^
g5103	Related to inhibitor of calcineurin	2.41E-114	86.65	−2.28	−1.19	2.30 × 10^−85^
g13846	Aminotriazole resistance protein	0	60.56	−2.64	−1.40	7.04 × 10^−24^
g10436	Subtilisin-like proteinase Mp1	1.38E-164	69.22	−2.96	−1.57	2.13 × 10^−4^
g5443	Hypothetical protein DL763_001348	1.90E-49	60.11	−6.03	−2.59	0.0046
g6520	Hypothetical protein NECHADRAFT_67887	1.14E-67	73.96	−7.42	−2.89	6.48 × 10^−4^
g7560	Hypothetical protein	0	58.51	−7.89	−2.98	3.57 × 10^−4^

* The table is arranged from most to least differentially overexpressed gene (highest fold change and log_2_FC to least).

**Table 4:** Putative ergosterol biosynthesis genes in *Che* and *Cps* identified using a GO term search.

Name	Description (Blastp Result)	Min *e*-Value	*Cps* % Identity	*Cps* Genes
*GO Name-P:ergosterol biosynthesis process*				
g6498	NADH-cytochrome b5 reductase 2	0	100	g4129
g133 *	cytochrome P450 61	1.1E-166	100	g3336
g7816	e3 ubiquitin-protein ligase hula	0	99.92	g5528
g13550	putative squalene synthase	0	99.86	g3858
g1530	cytochrome P450 61	0	99.69	g3336
g12864	NADPH-cytochrome P450 reductase	0	99.50	g9924
g9740	phytoene dehydrogenase	0	99.08	g12006
g5865	probable sterol C-24 reductase (ERG4)	0	99.02	g5534
g3859	NADPH-cytochrome P450 reductase	0	75.25	g9924

* This gene contained no predicted protein domains but shares 100% similarity with *Che* g1530 and *Cps* g3336, aligning only to the first 108 amino acids of both genes.

**Table 5:** Putative ergosterol biosynthesis genes in *Che* and *Cps* identified by *Fusarium* blastp.

Name	Description (Blastp Result)	*Fusarium* GenBank Accession	*Cps* Genome Multi-Blast		
			Min *e*-Value	*Cps* % Identity	*Cps* Genes
g10523	Acetyl-CoA acetyltransferase (ERG10)	TVY72719.1	0	99.92	g3826
* g9796	3-hydroxy-3-methylglutaryl-coenzyme A reductase (HMGCR)	CAA63970.1, sp|P0CT44.1|	0	99.72	g7570
g9170	mevalonate kinase (ERG12)	TVY77487.1	0	99.67	g10401
g13304	predicted protein (ERG8)	TVY72023.1	0	99.92	g9882
g11758	isopentenyl-diphosphate Delta-isomerase (IDI)	EWY96464.1	0	99.87	g9857
g6524	Farnesyl pyrophosphate synthase (FPS)	sp|S0E627.1|	0	99.29	g9279
g2103	Farnesyl pyrophosphate synthase (FPS)	sp|Q92235.1|	0	99.81	g10129
g13550	squalene synthase (ERG9)	EWY95524.1	0	99.86	g3858
g13589	squalene monooxygenase (ERG1)	TVY72423.1	0	99.87	g9959
g13485	geranylgeranyl pyrophosphate synthase (GPS)	sp|Q92236.1|	0	99.59	g2678
g13972	lanosterol synthase (ERG7)	TVY78064.1	0	99.75	g10346
* g8604	delta(14)-sterol reductase (ERG3 or ERG24)	ABB48844.1, TVY78591.1	0	99.73	g12760
g7626	methylsterol monooxygenase (ERG25)	TVY72244.1	0	99.89	g9698
g2642	3-keto-steroid reductase (ERG27)	TVY72041.1	0	99.72	g3656
g13957	predicted protein (ERG28)	TVY63301.1	0	99.61	g6640
g12891	C-8 sterol isomerase (ERG2)	EWY99288.1	0	99.85	g7224
g12992	cytochrome P450 61 (ERG5)	TVY77434.1	0	99.81	g6026

* Note that g8604 and g9796 closely matched with two distinct *Fusarium* ergosterol biosynthesis genes. The above data are arranged according to the pathway laid out by [17].

## Data Availability

Previously generated genome assemblies for *Cps* CT1 and *Che* CB45 are archived at the publicly accessible U.S. Department of Agriculture Ag Data Commons database at: https://data.nal.usda.gov/ (accessed on 1 January 2019).

## Supplementary Materials

The following are available online at https://www.mdpi.com/article/10.3390/ijms22179255/s1.
